# Books and Magazines

**Published:** 1898-09

**Authors:** 


MESSRS. WILLIAM WOOD & CO., of New York, have recently published the following list of important and timely books:

Handbook for the Hospital Corps of the U. S. Army and State Military Forces. By Charles Smart, Deputy Surgeon-General, U. S. A. Approved by the Surgeon-General of the Army. Second revised edition. 12mo, 358 pages, illustrated, blue muslin, $2.25 net.

Tropical Diseases. A Manual of Diseases of Warm Climates. By Patrick Manson, M. D., LL. D. (Aberd.), London. One volume of 625 pages, 12mo, illustrated by 88 wood-engravings and two colored plates. Muslin, $3.50 net.

Wounds in War. The Mechanism of Their Production and Their Treatment. By Surgeon-Colonel W. F. Stevenson, Professor of Military Surgery, Army Medical School, Netley, England. One volume of 588 pages, 8vo, profusely illustrated, muslin, $4 net.

A Manual of Surgery. For Students and practitioners. By William Rose, M. B., B. S. Lond., F. R. C. S., and Albert Carless, M. S. Lond., F. R. C. S. One volume, 8vo, of 1162 pages, very profusely illustrated by wood-engravings. Extra muslin, $5 net; leather, $5.75 net.

---

THE LITTLE QUEEN'S PICTURE.—Wilhelmina, who is to be crowned Queen of the Netherlands on September 6 next, has personally sent to Mr. Bok, the editor of *The Ladies' Home Journal*—himself a Hollander by birth—one of her private portraits for publication in the next number of his magazine. It is the last portrait which will be taken of the little lady before her coronation, and will be printed in connection with a specially prepared sketch, showing the personality of the first Queen of Holland from every point of view.

---

DR. MORITZ BUSCH, who has been sometimes described as Bismarck's Boswell, and who enjoyed terms of special intimacy with the great Chancellor, is the author of an important paper on Bismarck and William I., which will be published entire in *The Living Age* of Sept. 3. It was written with a view to publication after Bismarck's death and it contains so much that was communicated to the author by Bismarck himself that it is almost autobiographic.

